# The Regulation of CIN-like TCP Transcription Factors

**DOI:** 10.3390/ijms21124498

**Published:** 2020-06-24

**Authors:** Jingqiu Lan, Genji Qin

**Affiliations:** 1 State Key Laboratory of Protein and Plant Gene Research, School of Life Sciences, Peking University, Beijing 100871, China; lanjq@pku.edu.cn; 2 School of Advanced Agricultural Sciences, Peking University, Beijing 100871, China

**Keywords:** CIN-like TCP transcription factors, regulation, light, high temperature, microRNA319, BRAHMA, TIE1 transcriptional repressors, TEAR1 E3 ligases

## Abstract

TEOSINTE BRANCHED1/CYCLOIDEA/PROLIFERATING CELL FACTOR 1 and 2 (TCP) family proteins are the plant-specific transcription factors extensively participating in diverse developmental processes by integrating external cues with internal signals. The roles of CINCINNATA (CIN)-like TCPs are conserved in control of the morphology and size of leaves, petal development, trichome formation and plant flowering. The tight regulation of CIN-like TCP activity at transcriptional and post-transcriptional levels are central for plant developmental plasticity in response to the ever-changing environmental conditions. In this review, we summarize recent progresses with regard to the function and regulation of CIN-like TCPs. CIN-like TCPs are regulated by abiotic and biotic cues including light, temperature and pathogens. They are also finely controlled by microRNA319 (miRNA319), chromatin remodeling complexes and auxin homeostasis. The protein degradation plays critical roles in tightly controlling the activity of CIN-like TCPs as well.

## 1. Introduction

Developmental plasticity is central for sessile plants in adaptation to the environmental conditions [1]. The molecular bases for plant developmental plasticity or the mechanisms by which plants translate the environmental cues into the internal signals to direct the optimal growth and development in different plant growing conditions are important for plant survival and are useful for crop improvement by molecular breeding. Since the discovery of the founding members of TEOSINTE BRANCHED1/CYCLOIDEA/PROLIFERATING CELL FACTOR 1 and 2 (TCP) protein family in plants more than twenty years ago [2,3,4], TCP proteins have emerged as a central hub for integrating the internal and external cues to control plant developmental plasticity.

TCP is an acronym of the name of founding genes isolated from three species, i.e., *TEOSINTE BRANCHED1* (*TB1*) from maize (*Zea mays*) [2,5], *CYCLOIDEA* (*CYC*) from snapdragon (*Antirrhinum majus*) [3], and *PROLIFERATING CELL FACTOR 1* and *2* (*PCF1* and *PCF2*) from rice (*Oryza sativa*) [4]. *TB1* is a famous maize domestication gene. TB1 represses the outgrowth of axillary branches and promotes the formation of female inflorescences in domesticated maize, while in teosinte—which is the wild ancestor of maize—the twice lower expression of *TB1* leads to a decrease of apical dominance and an increase of shoot branches [5]. The *CYC* gene was isolated from snapdragon. *CYC* is specifically expressed in the dorsal primordia and controls the flower zygomorphic trait. Disruption of both *CYC* and its close homolog *DICHOTOMA* (*DICH*) in snapdragon results in radially symmetric flowers [6]. Both TB1 and CYC play pivotal roles in shaping plant key morphologies. The rice PCF proteins were found to directly bind to the promoter region of *PROLIFERATING CELL NUCLEAR ANTIGEN* (*PCNA*) gene which encodes a protein acting as a DNA polymerase sliding clamp implicated in DNA replication and cell cycle regulation [4]. Further analysis of the protein sequences of TB1, CYC and PCF proteins found that they all contain a conserved region predicted to form a non-canonical basic helix-loop-helix (bHLH) structure named as the TCP domain [7]. Since PCF1 and PCF2 had DNA-binding activity, TCP proteins were deduced to act as transcription factors and the TCP domain was proposed to be responsible for DNA binding and protein-protein interaction [4,7,8].

**Figure 1:**
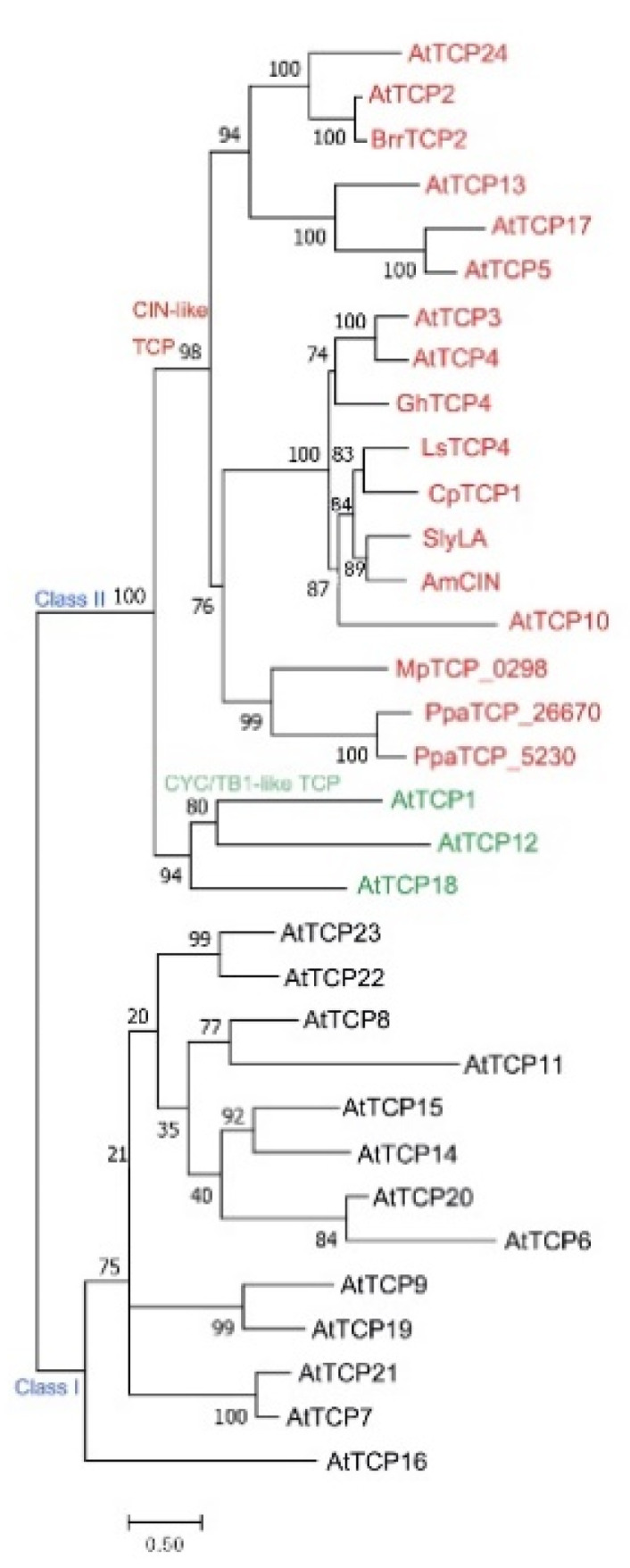
The phylogeny of TCP transcription factors, including all the TCP proteins in *Arabidopsis thaliana* and the CIN-like TCPs of other species mentioned in this review. Multiple alignments of the full-length TCP proteins were conducted using MAFFT Version 7 [15] with L-INS-i iterative refinement methods. The phylogenetic tree was constructed with the Maximum Likelihood (ML) method using the IQ-tree2 software [16] with the VT+F+R4 model with 1000 bootstrap replications. The subfamilies and subclasses (Class I, Class II, CIN-like TCP and CYC-like TCPs) are indicated above the divergent branches. The proteins in red words are the CIN-like TCPs which are mainly discussed in this review. The prefixes of TCP proteins are indicated the species. At: *Arabidopsis thaliana*; Brr: *Brassica rapa*; Gh: *Gossypium hirsutum*; Ls: *Lactuca sativa*; Cp: *Cyclamen persicum*; Sly: *Solanum lycopersium*; Am: *Antirrhinum majus*; Mp: *Marchantia polymorpha*; Ppa: *Physcomitrella patens*. The bootstrap support is indicated above the branches. The scale bar denotes the branch length.

**Figure 2:**
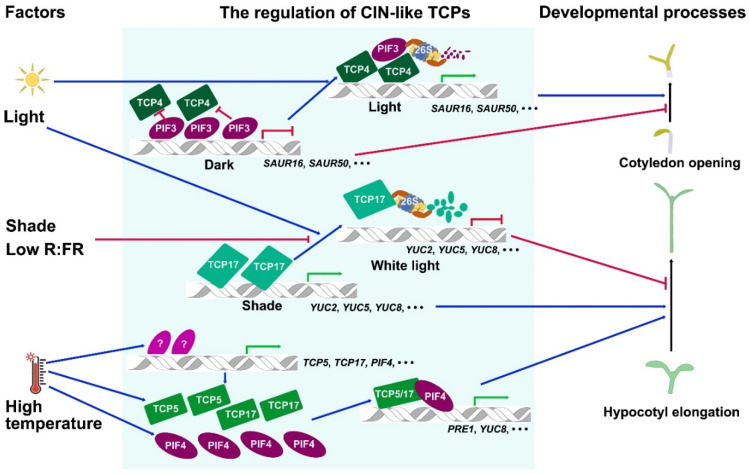
An overview of the regulation mechanisms of CIN-like TCP transcription factors by light and temperature during cotyledon opening and hypocotyl elongation processes. The external stimuli including light and high temperature are summarized at the left column. The schematic diagram includes the regulation mechanisms of CIN-like TCPs at the transcriptional and the protein levels. The arrows directly pointing on the double helix symbols indicate transcriptional regulations. The arrows pointing to the proteins indicate the regulations of protein stabilities. The proteins related with the “26S” symbols indicate protein degradation through the ubiquitin-26S proteasome pathway. The blue arrows represent the positive regulation, and the red arrows with dash-headed ends indicate the negative regulation. The green arrows and red dash-headed ends at the double-helix icons indicates the activation and repression of gene expression, respectively. All the unknown factors are indicated with question marks. R:FR, red light: far red light ratio; PIFs, PHYTOCHROME-INTERACTION FACTORs; SAURs, SMALL AUXIN UPREGULATED RNAs; YUCs, YUCCAs.

According to the sequence differences in the TCP domain, TCPs are classified into class I and class II subfamilies [7] (Figure 1). The TCP domain of class II TCPs contains additional four-amino acid residues in the conserved basic region [7]. The class II TCPs are further divided into CINCINNATA (CIN)-like TCPs and CYC/TB1-like TCPs based on the additional sequence differences in the TCP domain [9]. The CYC/TB1 TCP subgroup also carries a conserved glutamic acid-cysteine-glutamic acid (ECE) motif outside the TCP domain [10]. The *CIN* gene was isolated from snapdragon by analyzing the *cin* mutant which produces abnormal leaves and petals with undulated edges [11,12] and is the founding member of the CIN-like TCP subgroup (Figure 1). CIN controls leaf flatness by tightly regulating cell proliferation and differentiation in the different areas of leaf blades [11]. In the model plant *Arabidopsis*, the CIN-like TCPs include eight members which are further grouped into two clades based on the existence of microRNA (miRNA) binding site outside the sequence encoding TCP domain. *TCP2*, *TCP3*, *TCP4*, *TCP10,* and *TCP24* have the miRNA binding sites and post-transcriptionally regulated by miR319 [13], while TCP5, TCP13 and TCP17 form a small clade named as TCP5-like CIN-TCPs that were proved to be important for plant thermomorphogenesis (Figure 2) [14].

TCP transcription factors constitute a plant-specific protein family which is conserved in plant kingdom. TCP homologs are identified from diverse plant species [7]. It is proved that TCP proteins are existed in the early land plants during evolutionary history [17,18,19,20]. However, it is still in dispute whether they are present in pluricellular green algae [17,20]. The TCP protein family is significantly expanded in angiosperm species by gene or whole-genome duplication independently in basal angiosperm, magnoliids, basal eudicot, monocot, and many major groups within eudicot [10,17,20,21,22,23,24,25]. It is hard to distinguish whether class I or class II subfamily is the first to appear in plant kingdom, because the genome of liverwort *Marchantia polymorpha* contains the members belonging to both of the two families [19,26]. As for class II TCPs, the CIN-like TCP subgroup is predicted to be more ancestral than the CYC/TB1-like TCPs, since the class II TCPs all belong to CIN-like TCP subgroup in the non-vascular plants [18,19,26,27]. The CYC/TB1-like TCP group is proposed to originate in angiosperm species and to evolve independently in basal eudicot groups and monocot species [23,24,28,29,30,31].

TCP family transcription factors governs various key developmental processes during the life cycle of plants. TCPs regulate seed germination, leaf development, outgrowth of shoot branches, flowering, flower development, silique and ovule development, photomorphogenesis, thermomorphogenesis, circadian rhythms, defense responses and senescence [11,32,33,34,35,36,37,38,39,40,41,42,43,44,45,46,47,48,49,50,51,52,53]. The tight regulation of TCPs is very important for plant development and survival. Plants evolve many ways to tightly regulate TCP activity. The aim of this review is to give a comprehensive overview on current knowledge relevant to the roles of CIN-like TCPs in different species and the fine regulation of CIN-like TCP by external stimuli, miRNA and other proteins. To understand the detailed functions of TCPs in plants, the downstream targets regulated by TCPs, the regulation of CYC/TB1-like TCPs, please refer to the excellent recent reviews [39,54,55].

## 2. The Functions of CIN-Like TCP Transcription Factors in Different Species

One of the most prominent roles of CIN-like TCP transcription factors is that they play a conserved and central role in control of leaf flatness, size, shape and complexity. The loss of *CIN* function in snapdragon *cin* mutant disrupted leaf flatness and forms defective simple leaves with larger size and wavy margins [11,12,56]. In *Arabidopsis*, CIN-like TCPs have highly redundant and additive roles in regulating the morphogenesis of simple leaves (Figure 2). The *Arabidopsis tcp* single mutants produced leaves with no obvious differences from wild-type control. However, disruption of *TCP4* and *TCP10* had already led to larger and curled leaves. The high-order multiple *CIN*-like *tcp* mutants caused even severer leaf curvature and wavier leaf margins in a dose-dependent manner [53,57,58,59], indicating that the activity of CIN-like TCPs is pivotal for shaping leaf forms. The *CIN*-like *TCP* homolog in turnip (*Brassica rapa*), *BrrTCP2*, has conserved function in control of leaf size and morphology. Overexpression of *BrrTCP2* reduced the leaf size of wild-type *Arabidopsis* and restored the leaf morphology of the *Arabidopsis* multiple mutant *tcp2 tcp4 tcp10* [60]. In the regulation of leaf morphology, CIN-like TCPs repress the activity of leaf marginal meristem which determines leaf serrations in simple leaves or complexity of compound leaves in different plants. In lettuce (*Lactuca sativa*), the Empire type cultivars have more serrated leaves than the Salinas type cultivars. The molecular base is that Empire type cultivars carry a retrotransposable element inserted in the upstream of *LsTCP4* gene, causing lower expression level of *LsTCP4* than that in the Salina type cultivars. The downregulation of *LsTCP4* by the insertion led to the severer leaf serration in Empire type cultivars [61]. However, differential expression analysis between broad- and curly-leaved plants of *Cichorium endivia*, a close relative of *L. sativa* that also displayed wavy or serrated leaves, did not identify *TCP4-like* homologous genes as differentially expressed in leaves with different morphologies, and the two transcripts were abundant in both leaf types [62]. Tomato forms compound leaves regulated by *LACEOLATE* (*LA*) homologous to *CIN-*like *TCPs*. Downregulation of *LA* generated more and larger leaflets, causing super-compound leaves. On the contrary, overexpression of *LA* resulted in the compound leaves turning into simple leaves [63,64,65]. *CpTCP1* in cyclamen (*Cyclamen persicum*) is a homolog of *CIN-*like *TCPs*. Disruption of TCP function by a dominant repressor in which the ethylene-responsive element binding factor-associated amphiphilic repression (EAR) repression domain (SRDX) was fused to CpTCP1 caused irregular protrusions of acicular and branched shapes in the leaf margins [66]. CIN-like TCPs also regulate the leafy head of Chinese cabbage (*Brassica rapa*). Altering the spatio-temporal expression patterns of *BrpTCP4* led to a cylindrical head shape from a round one [67]. Furthermore, the genetic manipulation of CIN-like TCP activity resulted in different sizes and shapes of leaves in both simple and compound leaves [64,68]. These findings indicate that CIN-like TCPs are central regulators of leaf morphology and that the tight control of the spatio-temporal TCP activity is fundamental in determining diverse leaves in different species. 

CIN-like TCPs also modulate the development of organs homologous to leaves such as petals. The *Arabidopsis* single mutant *tcp5* produces wider petals than the wild-type control [69]. Moreover, the *35S:miR-3TCP* transgenic plants in which an artificial miRNA targeting to *TCP5*, *TCP13*, and *TCP17* was expressed to knock down the three genes generate petals with even increased width from tip to base [69]. Besides *TCP5-*like *CIN*-*TCPs* which was identified to determine petal size, the other five *CIN*-like *TCP* genes targeted by miRNA319 also played vital roles in control of petal growth. The mutant carrying a loss-of-function mutation in *miR319a* (named as *MiR319a^129^*) exhibited narrow petals and sterile anthers, indicating that CIN-like TCPs not only inhibit the growth of petal [70], but also play an essential role in plant fertility. The overexpression of the miR319-resistant form of *TCP4* by a petal-specific promoter rescued the narrow petals in *MiR319a^129^* mutant [70,71]. CIN-like TCPs also modify the morphology of petals besides petal sizes. Expression of a dominant repressor in which TCP3 was fused to an EAR motif to disrupt the function of TCPs resulted in curled petals in *Arabidopsis*. Expression of other CIN-like TCP chimeric repressors also caused curled petals [72]. The function of CIN-like TCPs in regulating petal development is conserved among different species. For examples, the introduction of chimeric repressors of *Arabidopsis* CIN-like TCPs in *Chrysanthemum morifolium* or *Ipomoea nil* also led to similar wavy and serrated petals [73,74]. Suppression of TCP functions by expression of chimeric repressors of CpTCP1 homologous to *Arabidopsis* TCP3 in *C. persicum* caused ruffled petals [66].

At the cellular level, CIN-like TCPs regulate cell proliferation, cell elongation or expansion and cell differentiation. During leaf and petal development, CIN-like TCPs inhibit cell proliferation and promote cell differentiation. Disruption of CIN-like TCPs prolong the leaf cell proliferation in the leaf blade with more rapid growth in the margin than in the center of blade, leading to the increased number of pavement cells and wavy margins [11,12,35,58,59,75]. As specialized epidermal cells, trichomes are also regulated by CIN-like TCP transcription factors. CIN-like TCPs suppress the trichome differentiation and subsequent trichome branching. The numbers of trichomes and trichome branches were both significantly increased in *jaw-D* and *tcp2 tcp4 tcp10* mutants, but were decreased in *TCP4* overexpression lines [76]. The function of CIN-like TCPs is also conserved in the regulation of trichome formation. Overexpression of *miR319a* in *Populus tomentosa* resulted in higher density of trichomes on the leaf surface when compared with that of wild-type control. When the functions of CIN-like TCPs were enhanced by inhibiting the roles of miR319, the number of trichomes was largely decreased [77]. Cotton fibers are specific trichome types on the seed epidermis. The constitutive overexpression of *GhTCP4* homologous to *Arabidopsis* TCP4 in upland cotton (*Gossypium hirsutum*) repressed the elongation of cotton fiber [78]. However, CIN-like TCPs positively regulate hypocotyl cell elongation in *Arabidopsis*. Induction of CIN-like TCPs using mTCP4-GR in which TCP4 fusion with rat glucocorticoid receptor (GR) by dexamethasone (DEX) treatment in transgenic lines significantly increased the length of hypocotyl cells (Figure 2) [79]. Overexpression of *TCP5-*like *CIN-TCPs* led to the significant increase of hypocotyl under shade, high temperature or under normal growth conditions (Figure 2) [14]. In consistence with the results, the *tcp5 tcp13 tcp17* triple mutant displayed short hypocotyls [14]. These findings demonstrate that CIN-like TCPs control cell proliferation, elongation and differentiation in a specific cell type-dependent manner at different context. 

CIN-like TCPs are reported to be essential for regulating other biological processes. For examples, CIN-like TCPs facilitate the transition from vegetative to reproductive growth. The flowering time of *cin-*like *tcp* multiple mutants was significantly postponed, while overexpression of *TCP4* led to early flowering in *Arabidopsis* [80]. The tomato *LA* gene belonging to *CIN-*like *TCP* group controls flowering as well [81]. In addition, CIN-like TCPs participate in developmental plasticity in response to biotic stresses in *Arabidopsis* and rice. CIN-like TCPs are also implicated in the typical morphological alterations caused by infection of phytopathogens such as phytoplasmas in *Arabidopsis* [43,44,45]. Rice ragged stunt virus (RRSV) downregulated rice *TCP21* belonging to miR319-targeted CIN-like TCPs by up-regulating the expression of *miR319* gene. Overexpression of *TCP21* increased the rice resistance to RRSV [82]. 

## 3. Light Regulates CIN-Like TCP Transcription Factors

Light is a critical environmental stimulus affecting plant development and growth including cotyledon opening, hypocotyl elongation and flowering [83,84,85,86]. When seeds germinate in dark under soil and then the seedlings grow out with exposure to light in nature, plants undergo an important morphological change from skotomorphogenesis to photomorphogenesis including cotyledon opening and inhibition of hypocotyl elongation [83,87,88]. The bHLH transcription factors PHYTOCHROME-INTERACTING FACTORs (PIFs) including PIF3 are central regulators in promoting skotomorphogenesis by suppressing cotyledon opening and the elongation of hypocotyl [89,90]. However, the molecular mechanisms of light-induced cotyledon opening are not well-known. Recently, CIN-like TCPs have been identified to participate in controlling light-induced cotyledon opening during photomorphogenesis (Figure 2). Interestingly, *CIN*-like *TCP* genes including *TCP3*, *TCP4* and *TCP10* are predominantly expressed in cotyledons under both light and dark growth conditions [13]. Why do CIN-like TCPs promote cotyledon opening in the light but not affect cotyledon closing in dark? Chromatin immunoprecipitation sequencing (ChIP-seq) and RNA sequencing (RNA-seq) analyses showed that TCP4 directly bind to the promoter regions of *SMALL AUXIN UPREGULATED RNA* (*SAUR*) genes including *SAUR16* and *SAUR50*. The promoter regions of the *SAUR* genes are also directly targeted by PHYTOCHROME INTERACTING FACTOR3 (PIF3), a key component inhibiting cotyledon opening. The molecular mechanism is that the accumulated PIF3 in the dark represses the transactivation activity of TCP4 possibly by competing the binding to the promoter regions of *SAUR* genes with TCP4 in the dark, while in the light PIFs are rapidly degraded and causing more TCP4 proteins to bind to the promoters of the *SAUR* genes and to upregulate their expression to promote cotyledon opening (Figure 2) [86]. However, PIF3 does not interact with TCP4 in this process. The exact mechanism by which PIF3 inhibits TCP4 binding to the promoter regions of *SAUR* genes is still an open question.

In addition to controlling the light-regulated cotyledon opening in plant photomorphogenesis, CIN-like TCPs also participate in the regulation of light-regulated hypocotyl elongation under shade. The shade avoidance syndrome (SAS) of plants caused by neighboring shade or low ratio of red light to far red light (R:FR) includes long hypocotyl, elongated leaf petiole, reduced shoot branches and early flowering [91]. It is known that shade or low R:FR upregulates the expression level of *BRC1* or *TB1* belonging to *CYC*/*TB1*-like *TCP* subgroup [37], while recently TCP5-like CIN-TCPs has been reported to regulate the rapid growth of hypocotyl in response to shade (Figure 2) [92]. The hypocotyl elongation of the triple mutant *tcp5 tcp13 tcp17* was insensitive to shade, while overexpression of *TCP17* led to longer hypocotyls under shade or white light. TCP17 is an unstable protein which is stabilized by shade. When plants were transferred from shade to white light, TCP17 was degraded and the degradation were inhibited by treatment with the 26S proteasome inhibitor MG132 [92]. This result indicates that white light promotes the degradation of TCP17 via the 26S proteasome, while shade inhibits the process (Figure 2). Interestingly, the transcriptional level of *TCP17* was rapidly downregulated by shade in reverse, indicating accumulation of TCP17 under shade is dependent on the post-transcriptional regulation [92]. It will be very interesting to identify the E3 ligase mediating the degradation of TCP17 under white light and the molecular mechanisms of suppression of the TCP17 degradation machinery by shade. 

## 4. High Temperature Regulates CIN-Like TCP Transcription Factors

Ambient temperature is one of the most important environmental factors governing plant behavior. Plants adopt a series of morphological changes called thermomorphogenesis in adaptation to high temperature [93,94]. Thermomorphogenesis includes leaf hyponastic growth, petiole elongation and hypocotyl elongation [93]. TCP5-like CIN-TCPs have recently been identified to act as key factors in positively regulating plant thermomorphogenesis. High temperature not only induces the expression of *TCP5, TCP13* and *TCP17* genes at the transcriptional level, but also stabilizes the protein of TCP5-like CIN-TCPs at the post-transcriptional level in *Arabidopsis* (Figure 2) [14,95]. Interestingly, high temperature treatment regulates both the expression level and the expression pattern of *TCP5*. When TCP5pro-GUS transgenic lines in which *GUS* reporter gene was driven by *TCP5* promoter was treated under high temperature, the GUS staining was strengthened in the hypocotyls and cotyledons, and at the same time was shifted from the leaf blades to petioles, in consistence with the leaf trait of thermomorphogenesis with elongated petioles and reduced areas of blades [14]. High temperature also up-regulates the expression of *PIF4* which is the first key factor identified in control of plant thermomorphogenesis [96,97]. TCP5 protein not only directly bound to the promoter region of *PIF4* gene to increase its expression level [14], but also interacted with PIF4 at the protein level [14]. Moreover, TCP17 protein interacted with the blue light receptor CRYTOCHROME1 (CRY1) at lower temperature to block the activity of TCP17. High ambient temperature increased the protein stability of TCP17 and led to the release of TCP17 from TCP17-CRY1 complex, promoting the interactions between TCP17 and PIF4 [93]. The interactions between PIF4 with TCP5 or TCP17 synergistically promoted the expression of a lot of common downstream genes including *PRE1* and *YUC8*, thus enhancing plant thermomorphogenesis (Figure 2) [14,95]. Accordingly, overexpression of *CIN-like TCP5* gene led to constitutive thermomorphogenesis, while the hypocotyls and petioles of *tcp5 tcp13 tcp17* were shorter than that of wild-type control under normal temperature or high temperature [14,95]. It is worth mentioning that although PIF4 is homologous to PIF3 which is a key regulator in photomorphogenesis [86], they use different mechanisms to regulate the activity of CIN-like TCPs. PIF3 do not interact with TCP4, but inhibits the binding activity of TCP4 to the promoter of their downstream genes in an unknown way under dark [86]. Adversely, PIF4 interacts with TCP5-like CIN-TCPs and obviously strengthened their transactivation activity in activating the downstream genes [14,95]. These results demonstrate that high temperature regulates the function of TCP5-like CIN-TCPs which positively regulate plant thermomorphogenesis by a different mechanism underlying the regulation of cotyledon opening by TCP4 in *Arabidopsis*. However, the transcription factors and E3 ligases that are responsible for regulating the expression of *TCP5*-like *CIN-TCPs* and the stability of their products under different ambient temperatures need to be further identified.

## 5. Phytoplasmas Regulate CIN-Like TCP Transcription Factors

Phytoplasmas are phytopathogens transmitted by insects and infect a wide range of plant species, causing great economic losses in agriculture [47,98]. Like the most pathogens, phytoplasmas produce effectors to alter the host-pathogen interface in facilitating their growth during infection [47]. The effectors cause some typical changes of plant morphology including overgrowth of lateral branches, altered leaf shape and sterile flowers [98]. The aster yellows phytoplasma witches’ broom (AY-WB) strain infect a wide range of dicot and monocot species [47,48]. The secreted AY-WB protein 11 (SAP11) is a virulence nuclear effector with a nuclear localization signal at its N-terminus. Overexpression of *SAP11* in *Arabidopsis* produced serrate and wavy leaves almost identical to those of *jaw-D* and the multiple *cin*-like *tcp* mutants [48]. SAP11 interacts with CIN-like TCP proteins [48], leading to the TCP degradation which is not inhibited by the 26S proteasome inhibitor epoxomicin or protease inhibitor cocktail (Figure 2). This indicates that the SAP11-mediating TCP protein degradation is not through ubiquitin-26S proteasome pathway [48]. Because CIN-like TCPs positively regulate the expression of *LOX2* gene by directly binding to its promoter [53], the overexpression of *SAP11* caused the downregulation of the *LOX2* gene and reduced the production of jasmonic acid (JA) in both *Arabidopsis* and tobacco (*Nicotiana benthamiana*), facilitating the infection of phytoplasmas [47,49]. The SAP11 protein homologs in different phytoplasmas strains displayed the varied abilities in control of the stability of CIN-like TCPs. These strains include AY-WB, onion yellow strain M (OY-M), peanut pupurea witches’ broom (PnWB), *Candidatus* phytoplasmas mali (CaPM) [49]. When SAP11 homologs were co-expressed with CIN-like TCPs in tobacco, the abundance of TCP proteins were measured to determine the abilities of SAP11 proteins in mediating TCP degradation [49]. The results showed that SAP11_AYWB_ had the strongest ability to mediate the degradation of TCP2, TCP3, TCP4, TCP5, TCP10 and TCP24, while SAP11_CaPM_ only mediated the degradation of TCP2 and TCP10 with lower ability than SAP11_AYWB_. SAP11_PnWB_ and SAP11_OYM_ only exhibited a weak ability to destabilize TCP2 [45]. The SAP11 homolog from the Maize Bushy Stunt Phytoplasmas (SAP_MBSP_) have been shown to only interact with CYC/TB1-like TCPs, but not any members of CIN-like TCPs in maize [46]. Accordingly, the MBSP-infected maize showed overgrowth of tillers controlled by the CYC/TB1-like TCPs, but not had any effects on the morphology of leaves [46]. Similarly, SWP1 which is a SAP11-like phytoplasmas effector from wheat blue dwarf phytoplasma interacted with BRC1 and mediate the degradation of BRC1 when *SWP1* was overexpressed in *Arabidopsis* [50]. These findings indicate that the effector SAP11 proteins from different phytoplasmas strains have different specificity in promoting the degradation of TCPs. As the SAP11 protein have no protease activity, the mechanisms underlying SAP11-mediated TCP degradation remains to be further discovered [48].

## 6. miRNAs Regulate CIN-Like TCP Transcription Factors

**Figure 3:**
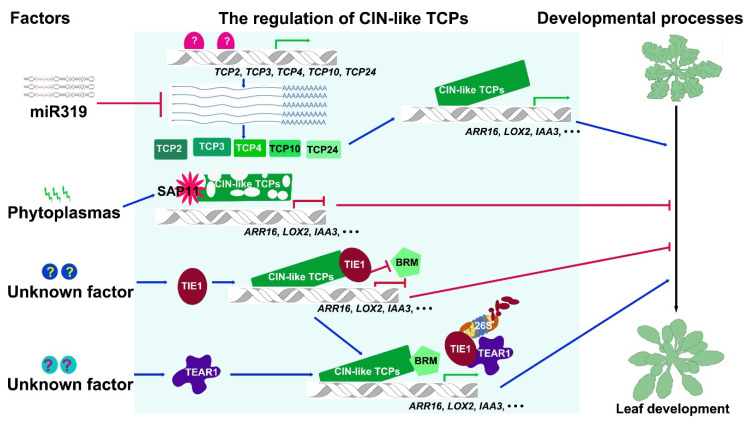
An overview of the regulation mechanisms of CIN-like TCP transcription factors during leaf development. The external stimuli and internal factors are summarized at the left column. The schematic diagram includes the regulation mechanisms of CIN-like TCPs at the transcriptional level, at the post-transcriptional level, and at the protein level. The arrows directly pointing on the double helix symbols indicate transcriptional regulations. The arrows pointing to the proteins indicate the regulations of protein stabilities or antagonistic functions. The proteins related with the “26S” symbols indicate protein degradation through the ubiquitin-26S proteasome pathway. The blue arrows represent the positive regulation, and the red arrows with dash-headed ends indicate the negative regulation. The green arrows and red dash-headed ends at the double-helix icons indicates the activation and repression of gene expression, respectively. All the unknown factors are indicated with question marks. R:FR, red light: far red light ratio; YUCs, YUCCAs; LOX2, LIPOXYGENASE 2; SAP11, SECRETED AY-WB PROTEIN 11; ARR16, ARABIDOPSIS RESPONSE REGULATOR 16; IAA3, INDOLE-3-ACETIC ACID INDUCIBLE 3; BRM, BRAHMA; TIE1, TCP INTERACTOR CONTAINING EAR MOTIF PROTEIN 1; TEAR1, TIE1-ASSOCIATED RING-TYPE E3 LIGASE 1.

miRNAs are small RNAs that recognize targeting mRNA via base pairing to the highly complementary binding sites and suppress the stability and translation of mRNAs [99,100]. A subset of *CIN-*like *TCP* genes contains a miR319-targeting sequence at the 3’-terminus of transcripts in almost all angiosperm groups [13,63]. The Arabidopsis *jagged and wavy*-*Dominant* (*jaw-D*) mutant was first identified from a collection of activation tagging mutants by forward genetics [13]. The mutant *jaw-D* displayed a predominant phenotype with the serrated and curved leaves [13,101]. Further analysis showed that T-DNA with four cauliflower mosaic virus (CaMV) 35S enhancer was inserted in neighboring region of MIRNA gene *MIR319a* in *jaw-D*. The expression of *miR319a* was activated and the target *CIN*-like *TCP* genes including *TCP2*, *TCP3*, *TCP4*, *TCP10* and *TCP24* were significantly downregulated in the mutant, suggesting that the transcript abundance of the corresponding *TCP* genes was regulated by miR319a (Figure 3) [13]. The overexpression of *miR319* also caused epinastic cotyledons, more trichomes, defective secondary cell wall biosynthesis and venation patterning, a modest delay in flowering, crinkled petals, short stamen, reduced male fertility and crinkled fruits by downregulating *CIN*-like *TCP* genes [42,70,102,103,104]. *Arabidopsis* genome contains three *MIR319* genes including *MIR319a*, *MIR319b*, and *MIR319c* which have highly redundant function in control of the abundance of *CIN*-like *TCP* transcripts [70]. However, the three *MIR319* genes also showed largely non-overlapping expression patterns revealed by GUS reporter analysis in plants, suggesting that they may have distinct roles in control of *TCP* abundance in a temporal and spatial manner during plant development [70]. During leaf development, the *MIR319a* gene is only expressed at the stipules, which is completely complementary to the expression pattern of *MIR319c* that the highest expression level is detected at the basal region of leaf primordia and young leaves, indicating the functional divergences between the two genes. *MIR319b* is only expressed in the sepal and stamen abscission zones of inflorescences at the reproductive stage [70]. *MIR319a* and *MIR319c* have partially spatiotemporal overlapping expression patterns during early inflorescence development [70]. Though the GUS activity for promoter analysis of *MIR319b* was not detected in leaves, the *mir319b* single mutant moderately reduced the size of leaf serrations, and *mir319a/b* double mutant almost entirely suppressed serration formation [59], suggesting that *MIR319b* is essential for leaf development with a possible low expression level in leaves.

miR319 is a conserved and ancient plant miRNA family and plays important roles in plant morphological adaptation to environmental conditions by targeting *TCP* for degradation. The miR319 and miR159 share highly similarity in mature miRNA sequence, secondary structure, conservation pattern and biogenesis in *Arabidopsis*. miR319 and miR159 are proposed to evolve from a common ancestor in land plants [105]. miR159 did not induce the cleavage of *TCP* mRNAs due to the specificity of sequences, while miR319 mediated the cleavage of *MYB33* and *MYB65* mRNA, which are pivotal targets of miR159 [101,105]. Two miR319 copies were identified in the genome of *M. polymorpha,* which also contains two *MpTCP* genes [106]. However, the two *MpTCP* genes have no possible miR319-targeting site and one target of miR319 was identified as *MpMYB33* [106,107]. In *Physcomitrella* and *Selaginella*, the *TCP* genes also have no miR319-targeting sites [108,109,110], indicating that miR319 regulation of *CIN*-like *TCP* possibly evolve after the divergence of lycophytes and euphyllophytes.

## 7. Chromatin Remodeling Complexes Regulate the Activity of CIN-Like TCPs

The activity of CIN-like TCPs is controlled by chromatin remodeling complexes including SWITCH/SUCROSE NONFERMENTING (SWI/SNF) complex and TCP INTERACTOR CONTAINING EAR MOTIF PROTEIN 1 (TIE1)-TOPLESS (TPL)/TOPLESS-RELATED (TPR) complex at the protein level (Figure 3). SWI/SNF complexes use ATPase to provide the energy in deciding the nucleosome position conformation and thus determining the accessibility of chromatin [111]. *BRAHMA* (*BRM*) encodes a SWI/SNF ATPase in *Arabidopsis* [112,113,114]. The hypomorphic mutations in *BRM* suppressed the phenotypes including fewer trichomes and smooth margins in *TCP4* overexpression lines [114]. And the hypomorphic *brm* mutants produced curled leaves and delayed leaf maturation resembling the multiple *cin*-like *tcp* mutants, indicating that BRM promotes the activity of CIN-like TCPs (Figure 3) [114]. BRM interacts with TCP4 and together bind to the promoter region of type A *ARABIDOPSIS RESPONSE REGULATOR* (*ARR*) gene *ARR16* to promote the expression of *ARR16* (Figure 3) [115]. The modulation of CIN-like TCP activity by BRM provides a fine regulation of leaf sensitivity to the phytohormone cytokinin (CK) during leaf development. 

Compared with the positive regulation of CIN-like TCP activity mediated by BRM, TIE1-TPL/TPR complexes repressed CIN-like TCP activity by recruiting histone deacetylases (HDA) (Figure 3) [116]. TIE1 was identified to regulate TCP activity by analyzing a gain-of-function mutant *tie1-D* obtained by screening a collection of activation tagging mutants for leaf-defective ones. Overexpression of *TIE1* in *tie1-D* or in transgenic plants using CaMV 35S promoter to drive *TIE1* all led to curled and serrated leaves that are observed in the multiple *cin*-like *tcp* mutants [116]. *TIE1* encodes a transcriptional repressor containing a typical EAR motif at the C-terminal end. Indeed, TIE1 has transcriptional repression activity and directly interacts with the corepressor TPL/TPRs through EAR motif. TIE1 also interacts with CIN-like TCPs via the N-terminal domain. Consequently, TIE1 suppresses the activity of CIN-like TCPs by acting as a bridge connecting corepressor TPL/TPRs with CIN-like TCPs during leaf development (Figure 3) [116]. Interestingly, TIE1 also interacted with BRC1 belonging to CYC/TB1-like TCP group [40]. *TIE1* had overlapping expression pattern with *BRC1* in young axillary buds and overexpression of *TIE1* resulted in excessive branches, indicating that *TIE1* also represses the activity of BRC1 during shoot branching [40]. The function of *TIE1* is conserved in controlling shoot branching in cotton (*Gossypium hirsutum*) [117]. GhTIE1 interacted with CYC subclade proteins GhBRC1, GhBRC2, and GhTCP13 *in vivo.* Silencing of *GhTIE1* in cotton seriously decreased shoot branching [117]. A similar mechanism in suppression of CIN-like TCP activity is mediated by SPOROCYTELESS/NOZZLE (SPL/NZZ) during ovule development [118]. SPL/NZZ is a key regulator responsible for promoting the differentiation of megasporocytes. No megasporocytes were formed in the ovules of *spl*/*nzz* mutants. SPL/NZZ also contains a typical EAR repressor motif at the C-terminal domain and has the transcriptional repression activity. SPL/NZZ uses C-terminal EAR motif to interact with TPL/TPRs and uses its N-terminal domain to interact with CIN-like TCPs [118]. Overexpression of *SPL* in T-DNA activation tagging mutant *spl-D* caused the defective ovule arrangement in ovaries resembling to that of the multiple *cin*-like *tcp* mutants. Consistently, overexpression of the *CIN*-like *TCPs* led to no megasporocytes resembling the phenotype of *spl* loss-of-function mutants [118]. These results indicate that SPL inhibits the activity of CIN-like TCPs in a way similar to TIE1 by connecting TPL/TPR corepressors with CIN-like TCPs. 

The regulation of CIN-like TCP activity by TIE1, SPL or BRM during leaf or ovule development is parallel to the regulation of key regulators in auxin signaling. The EAR motif-containing AUXIN (AUX)/INDOLE-3-ACETIC ACID (IAA) repressors mediate auxin signaling by recruiting TPL/TPRs to suppress the activity of AUXIN RESPONSE FACTORS (ARFs) [119,120,121]. Auxin triggers the degradation of AUX/IAA via 26S proteasome, the released ARFs such as MONOPTEROS (MP) bind to SWI/SNF chromatin remodeling ATPases BRM to promote the accessibility of chromatin and the expression of downstream genes. Interestingly, TIE1 is also an unstable protein as AUX/IAA repressors and the degradation of TIE1 is mediated by an E3 ligase TIE1-ASSOCIATED RING-TYPE E3 LIGASE1 (TEAR1) (Figure 3) [122]. Disruption of *TEAR1* leads to serrated and curled leaves similar to that observed in the multiple *cin*-like *tcp* mutants and *tie1-D* [122]. These findings suggest that *TEAR1* indirectly regulates the activity of CIN-like TCPs by switching the interactors of CIN-like TCPs from TIE1 to BRM (Figure 3), thus changing the chromatin state to control leaf development. However, the signals triggering the TIE1 degradation to release the suppression of CIN-like TCPs by TEAR1 need to be further identified.

## 8. Concluding Remarks and Perspectives

CIN-like TCPs are key transcription factors essential for plant growth and development in response to environmental cues and internal signals. The temporal and spatial activity of CIN-like TCPs determines cell proliferation, expansion and differentiation of cells in different organs in shaping plant morphology at various developmental stages. Consequently, the fine-tuning of CIN-like TCP activity is critical for plant developmental plasticity. At the transcriptional level, *CIN*-like *TCPs* are dynamically and specifically expressed in organs and also are induced by environmental signals including light and temperature [14,92,95]. However, the upstream regulation which determines the dynamic expression pattern and induction of *CIN*-like *TCP* genes are insufficient. The transcriptional repressor RABBIT EARS (RBE) has been reported to decreased the expression of *TCP4*, *TCP5*, *TCP13* and *TCP17* in promoting petal growth and *TCP4* and *TCP5* are possibly direct targets of RBE [69,71]. More studies on detailed analysis of the promoter regions of *CIN*-like *TCPs* are necessary for elucidating other upstream regulators, especially the direct regulators. The truncated promoters can be used to drive reporters in determining the minimal regions required for the expression patterns of *CIN*-like *TCPs*. Transcription factors directly interacting with the promoters of *CIN*-like *TCPs* could be identified by yeast-one-hybrid screening. 

CIN-like TCPs are central for regulating biosynthesis and signaling of different phytohormones including auxin, JA and brassinosteroid (BR) [53,123,124]. However, little is known about how phytohormones regulate CIN-like TCPs. It has been shown that auxin, gibberellin (GA), strigolactone (SL) and cytokinin (CK) regulate *BRC1* belonging to *CYC*/*TB1*-like *TCP* group of class II *TCPs* [36,125,126]. The decreased auxin level by overexpression of *IAA CARBOXYL METHYLTRANSFERASE1* (*IAMT1*) which converted IAA to methyl-IAA ester led to curly leaves and reduced the expression level of some *CIN*-like *TCPs* [127], indicating that auxin positively regulates *CIN*-like *TCPs* at the transcriptional level. Further studies are needed to determine whether other plant hormones and environmental signals except light and temperature could possibly regulate CIN-like TCPs and how these signals could be integrated to control the activity of CIN-like TCPs.

At the post-transcriptional level, the miR319-*TCP* regulation module is conserved and widely studied in several plant species [13,63]. Could the other miRNAs targeting *CIN*-like *TCPs* exist in different plant species? which are those transcription factors deciding the expression level and pattern of *MIR319* genes? These questions are still open.

At the protein level, we know little about the degradation mechanisms of CIN-like TCPs mediated by 26S proteasome or other protein degradation pathways. The regulation mechanisms of CIN-like TCPs by class I TCP transcription factors and other interacting proteins are still largely unknown. It is still a challenge to thoroughly understand the shaping of plant morphology controlled by the CIN-like TCP-centered network under various environmental and developmental conditions in *Arabidopsis* and the other plant species.
